# Feasibility and Preliminary Efficacy of a Foot-Ankle Exercise Program Aiming to Improve Foot-Ankle Functionality and Gait Biomechanics in People with Diabetic Neuropathy: A Randomized Controlled Trial

**DOI:** 10.3390/s20185129

**Published:** 2020-09-09

**Authors:** Renan L. Monteiro, Jane S.S.P. Ferreira, Érica Q. Silva, Asha Donini, Ronaldo H. Cruvinel-Júnior, Jady L. Verissímo, Sicco A. Bus, Isabel C.N. Sacco

**Affiliations:** 1 Departamento de Fisioterapia, Fonoaudiologia e Terapia Ocupacional da Faculdade de Medicina da Universidade de São Paulo, Rua Cipotânea, 51 - Butantã, São Paulo, SP 05360-000, Brazil; renanlm@usp.br (R.L.M.); janesuelen@usp.br (J.S.S.P.F.); ericaqueiroz10@usp.br (É.Q.S.); thales.panissa@usp.br (A.D.); ronaldocruvinel@usp.br (R.H.C.-J.); jady.verissimo@usp.br (J.L.V.); 2 Department of Physical Therapy, Federal University of Amapá, Rod. Juscelino Kubitschek, km 02 - Jardim Marco Zero, Macapá, AP 68903-419, Brazil; 3 Amsterdam UMC, University of Amsterdam, Department of Rehabilitation Medicine, Amsterdam Movement Sciences, Meibergdreef 9, 1105 AZ Amsterdam, The Netherlands; s.a.bus@amsterdamumc.nl

**Keywords:** plantar pressure, range of motion, diabetic neuropathies, exercise, diabetic foot, clinical trial, physical therapy

## Abstract

Foot-ankle strengthening and mobility exercises are part of international guideline recommendations for people at risk of diabetic foot disease. We examined the feasibility and preliminary efficacy of a 12-week foot-ankle exercise program on clinical, functional and biomechanical outcomes in people with diabetic neuropathy (DPN). We randomly allocated 30 people with DPN to usual care (control) or usual care plus a supervised exercise program (intervention). For feasibility, we assessed recruitment rate and participant adherence and satisfaction. For program efficacy, we assessed baseline to 12-week changes in daily physical activity level, gait speed, tactile sensitivity, ankle range of motion, DPN symptoms, quality of life, foot health and functionality, foot strength and plantar pressure during gait, using paired t-tests (*p* < 0.05). In 52 weeks, we recruited 45 eligible participants (0.90/week). Program adherence was 80% and participants’ satisfaction had a mean (SD) of 4.57 (0.70) out of 5. The intervention group significantly improved on toes strength, contact time during gait and DPN symptoms, and peak forefoot pressures increased over time; controls showed significantly increased heel peak pressures and force. The exercise program was feasible, based on a moderate recruitment rate and an adherent and satisfied population, and the intervention showed several positive preliminary effects over time compared to usual care.

## 1. Background

Diabetic neuropathy (DPN) is a symmetrical disorder, either clinically evident or subclinical, that occurs in people with diabetes; DPN is attributable to metabolic and microvascular alterations resulting from chronic hyperglycemia exposure as well as to cardiovascular risk covariates [1]. As DPN progresses, it affects the integrity of neural structures and, especially, small joints and intrinsic muscles of the foot-ankle [2,3,4,5,6]. These specific DPN consequences are the main factors for the development of foot deformities, increased plantar pressures during walking, and consequently the risk for plantar ulceration [2,7,8,9]. 

Supervised foot-related exercises combined with a health-promoting program were shown to effectively reduce DPN symptoms [10,11], improve vibration perception [12], recover foot and ankle joint mobility [12,13,14,15,16], redistribute pressure during walking [12,13,15,17,18,19,20] and increase foot strength and function [10,21,22]. All of these benefits mitigate the risk factors for foot ulceration in diabetes. Several foot-related exercises have been recommended in international guidelines to help reduce the incidence of foot ulceration in people at risk. However, because the quality of the evidence supporting the beneficial effects of foot-related exercises remains weak [23], physiotherapy interventions have not yet been implemented worldwide for preventing the progression of the musculoskeletal deficits in people with diabetes and DPN. For this reason, it is still unclear how the compliance with this type of preventive programs would be in this population and whether the recruitment for a trial that tests the efficacy of these physiotherapy programs would be feasible. 

Although the performance of exercises has been effective for improving the musculoskeletal health [17,20,24,25] and functional balance [21,26] of people with DPN, the recommended exercises usually target larger joints and muscles of the lower limbs and focus mainly on gait and balance training. These exercises do not address the specific musculoskeletal deficits of distal and smaller joints and muscles, which affect the functionality and biomechanics of daily living activities. Some of the most recommended foot-related exercises in the literature that have to be part of an intervention program are based on short foot exercises and Vele´s forward and reverse tandem gait exercise [10,27,28]. Therefore, more high-quality well-designed controlled trials are warranted to strengthen the level of evidence supporting the use of specific foot-ankle therapeutic exercises to mitigate the risk factors of, and help prevent, foot ulceration in people with diabetes.

A few randomized controlled trials (RCTs) evaluated the effects of foot-related strengthening and mobility exercises in different domains (i.e., clinical, functional, and biomechanical); the majority of these RCTs were of low quality, presented small effect sizes, and did not involve exercises that specifically target the main musculoskeletal dysfunction in people with DPN [23]. As regards the development of a guideline for foot ulcer prevention [29], only three studies [10,13,15] assessed the effects of foot-ankle exercise on DPN-related outcomes. All of this makes it difficult to design an RCT on this topic involving the investigated population and to determine what makes a preventive program relevant in terms of exercise inclusion, frequency and intensity of sessions, outcomes used and level of adherence and motivation to the program. Therefore, aspects of the feasibility and the preliminary efficacy of a more comprehensive training program should first be investigated.

The present work presents the results of a feasibility study involving a superiority RCT and preliminary efficacy analysis of a 12-week therapeutic foot-ankle exercise program for people with DPN [30]. It is also our intention with this paper to stress the use of biomechanical sensors to guide therapeutic strategies and rehabilitation of the dysfunctions related to DPN. These sensors are neither regularly applied to this population nor applied in clinical settings where this population is treated. Thus, besides the feasibility purposes, we aimed to emphasize the importance of the biomechanical sensor for trials that focus on therapeutic strategies for musculoskeletal deficits in the diabetic population. The parameters investigated were derived from biomechanical sensors and other clinical tools: foot functionality and clinical and biomechanical outcomes, such as gait speed, foot strength and plantar pressure during gait, as well as the aspects of recruitment, adherence to training protocol and participant satisfaction. 

Our hypotheses were as follows: (1) the program will be feasible and (2) the preliminary results of the intervention will show an increase in the toe and hallux isometric muscle strength, daily physical activity levels, self-selected and fast gait speeds, and passive ankle range of motion; an improvement in the foot tactile and vibration sensitivity, health-related quality of life and foot health and functionality; a decrease in the tactile sensory threshold and DPN symptoms; and an improvement in the foot rollover as represented by a more homogeneous plantar pressure distribution during gait.

## 2. Methods

### 2.1. Study Design and Ethics

**Figure 1:**
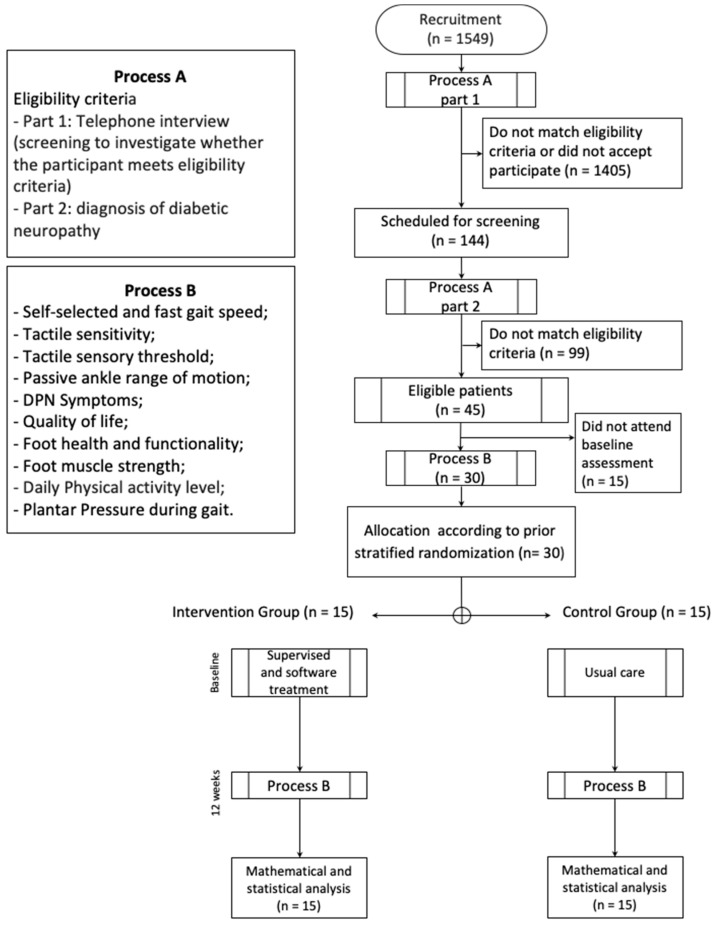
Flowchart of the feasibility study. DPN - Diabetic neuropathy.

Data for this feasibility study were collected between November 2017 and November 2018 (Figure 1) in the outpatient physiotherapy clinic of Centro de Saúde Escola Barra Funda Dr. Alexandre Vranjac, a primary care center; the assessments were performed at the physical therapy department of the School of Medicine of the University of São Paulo. All subjects gave their informed consent to participate in this study. The trial was conducted in accordance with the Declaration of Helsinki, was approved by the Ethics Committee of the School of Medicine of the University of São Paulo (Resolution 196/96 of the National Health Council; Research protocol No. 1.464.870), and was prospectively registered at ClinicalTrials.gov as NCT02790931. The research protocol has been published elsewhere [30].

### 2.2. Participants

The first 30 participants who were selected via convenience sampling were recruited, allocated, and completed the exercise program. Feasibility studies usually entail a smaller sample size compared with a full randomized trial as no formal calculation of power is required in the former [31]. Adults of up to 75 years of age with moderate or severe DPN were recruited through digital advertisement and through direct recruitment of people with diabetes during the health campaigns promoted by the State of São Paulo.

Individuals were eligible if they had type 1 or 2 diabetes mellitus; with moderate or severe DPN as diagnosed by the fuzzy decision support system [3]; able to walk independently for at least 10 m; with a maximum of one amputated toe, not being the hallux; and with Internet access that allows the use of a web-based software for the supervised exercise sessions. Individuals were excluded if they had the following: plantar ulcer; history of a surgical procedure in the knee, ankle, or hip or an indication of lower limb arthroplasty; the need to use a walking aid, such as a walker or cane; diagnosis of other neurological diseases besides DPN; dementia or inability to give consistent information; received any physiotherapy during the intervention period; diagnosis of a major vascular complication and/or severe retinopathy, as determined from their medical records.

The participants’ eligibility was checked by physiotherapists who were responsible for the outcome assessments in the trial and who were blinded to the treatment allocation. These physiotherapists collected the demographic, anthropometric, and clinical (history) data, as well as the data on foot and ankle function and plantar pressure during walking in a baseline assessment. At baseline, the participants were scheduled for a final assessment at the end of the 12-week follow-up period.

### 2.3. Randomization, Allocation and Blinding

The randomization scheme was prepared with the Clinstat software (University of York, York, UK) by an independent researcher who was blinded to the group allocation. A numerical sequence was placed in opaque envelopes that were numbered sequentially based on the order generated by the software. This sequence was kept private and stored in a location that is inaccessible to the blinded outcome assessors. Only the physiotherapist responsible for the supervised physiotherapy session was aware of the group allocation.

The participants’ personal data were kept confidential before, during and after the study through the assignment of a research code for each participant. Apart from the physiotherapist who was responsible for the randomization, the participant was also aware of his/her own code assignment. Two other physiotherapists, both blinded to the treatment allocation, were responsible for all clinical, functional, and biomechanical outcome assessments. The participants were instructed not to reveal their treatment allocation to the physiotherapist who conducted the assessments.

### 2.4. Intervention Protocol

The control group participants received the usual care recommended by medical staff and by the guidelines of the International Working Group on the Diabetic Foot (IWGDF) [29], as follows: (1) screening for a history of foot ulceration or lower-extremity amputation, peripheral artery disease, foot deformity, pre-ulcerative signs on the foot, poor foot hygiene, and ill-fitting or inadequate footwear; (2) inspecting the feet and the insides of shoes daily, washing the feet daily (with careful drying, particularly between the toes), avoiding the use of chemical agents or plasters to remove calluses or corns, using emollients to lubricate dry skin, and cutting toe nails straight across; (3) providing education aimed to improve foot care knowledge and behavior, as well as encouraging the participants to adhere to this foot care advice. All of these usual care orientations were given during the baseline session by the physiotherapist who conducted the study.

The intervention group participants received the usual care, along with a 12-week therapeutic exercise program that strengthens the muscles and improves the functionality of the foot-ankle complex. A part of the exercise protocol was performed twice a week under the supervision of a physiotherapist, and a series of foot and ankle exercises was performed twice a week by the participant alone, who was remotely supervised through the Educational Diabetic Foot Software (SOPeD, www.soped.com.br). The exercise protocol was designed to be as simple and practical as possible to effectively manage the musculoskeletal complications related to diabetes and to facilitate its implementation in primary and secondary public health care units. Both protocols (SOPeD and supervised therapeutic exercises) were designed to consist of the same set of modules: (a) warm-up exercises, (b) strengthening of the intrinsic foot muscles, (c) strengthening of the extrinsic foot muscles, and (d) functional exercises, such as balance and gait training. The SOPeD consisted of eight exercises that were divided into four modules. To promote long-term participation, each supervised session was conducted in groups of five to eight participants [26] with a minimum duration of 50 min. The exercise progression was customized to each patient as the supervised exercises were a face-to-face intervention that were executed according to the criteria set in physiotherapy programs. To avoid monotony and to increase motivation, the exercises were varied every session, and the maximum duration of each session was 20 min.

### 2.5. Outcomes

For the purpose of this study, the intervention was considered feasible based on the following criteria: (a) the adherence to the 12-week intervention protocol and to the final outcomes assessment was >80%; (b) the participant’s recruitment rate was close or equal to what the laboratory restrictions for the outcomes assessment require (10 participants/week); and (c) the participant satisfaction toward the intervention was >4 on a 5-point Likert scale. The preliminary efficacy of the training program was assessed based on whether the intervention group displayed significantly improved toe and hallux isometric muscle strength between baseline and after 12 weeks (T12). In the full RCT, the primary outcomes are the daily ambulatory activity level and self-selected and fast gait speeds, and the secondary outcomes are the foot isometric muscle strength, ankle joint range of motion, tactile sensitivity, DPN symptoms, quality of life, foot health and functionality and plantar pressure distribution during walking. 

#### 2.5.1. Outcomes for Feasibility

*Recruitment*

Recruitment was assessed in terms of recruitment rate and recruitment success. The recruitment rate is the ratio between the number of eligible individuals and the duration of the recruitment period (52 weeks); it is expressed as individuals per week. The challenges in recruitment were described qualitatively. Recruitment success is the ratio between the number of individuals who underwent baseline assessment and the number of eligible individuals who were contacted within the 52-week recruitment period.

*Adherence to the Exercise Program and to the Assessments and Dropout Rate*

The adherence to the foot and ankle exercise program is the percentage of participants who completed more than 80% of the 24 face-to-face sessions in 12 weeks. The dropout rate is the proportion of participants who terminated their participation in the therapeutic exercise program and dropped out of the study. The adherence to final outcomes assessment is the proportion of participants who had completed the T12 assessment.

*Participant Satisfaction*

The participant satisfaction with the therapeutic exercise program was evaluated by using a questionnaire at the end of the 12-week program. The items where the 5-point Likert scale was used included affirmative statements: (1) “I am satisfied with the exercise protocol”; (2) “The exercise protocol is easy to perform”; (3) “The exercise protocol is fun to perform”; (4) “The exercise protocol reached my expectations”; (5) “The exercise protocol somehow improved my walking practice”. The participants knew that their responses were anonymous, that is, the investigators do not know their identity. The score for each item ranged from 1 (strongly disagree) to 5 (absolutely agree), with the higher scores indicating greater participant satisfaction. 

#### 2.5.2. Outcomes of the Efficacy of the Exercise Program

Most of outcomes used to test the preliminary efficacy of the exercise program were derived from biomechanical sensors that have previously demonstrated their important clinical repercussions for individuals with DPN [23].

*Toe and Hallux Isometric Muscle Strength*

Toe and hallux isometric muscle strength was measured according to the method of Mickle et al. (2009) [32] wherein a pressure platform (emed q-100, Novel, Munich, Germany) was used. The subjects were asked to stand and push down on the platform two times and as hard as possible with their hallux and toes, which prevents excessive body sway. The maximum force under the hallux and toes normalized by bodyweight (BW) were the outcomes for this measurement. 

*Daily Physical Activity Level (Number of Steps)*

The level of daily physical activity was inferred from the number of steps taken for six continuous days as determined by using a 3D accelerometer (Power Walker-610, Yamax, Japan). This equipment measures the total number of steps and the distance covered, and it has been previously validated with older people and individuals with DPN [33,34].

*Self-Selected and Fast Gait Speeds*

In our gait lab, the participants first walked barefoot on a 10-meter track at a comfortable pace to determine their self-selected gait speed and then as fast as possible on the same track to determine their fast gait speed. For both speeds, two trials were conducted, and their average speed was calculated and used for analysis. Two photocells (CEFISE, Speed Test Fit Model, Nova Odessa, Brazil) located in the middle (at the 6 m mark) of the 10-meter walking track were used to measure the walking time and to calculate the gait speed. Gait speed is of great clinical value, as it is closely related to mortality; White et al. (2013) [35] have shown that older adults, as our participants, with fast decline in gait speed had a 90% greater risk of mortality than those with slow decline over time. Thus, even with a basic biomechanical sensor such as photoelectric cells, the outcome gait speed can be of paramount importance for monitoring the health status of DPN individuals.

*Plantar Pressure during Gait*

A 700 × 403 mm pressure platform (emed q-100) with 6080 sensors (4 sensors/cm^2^) that collects data at 100 Hz was used to assess the plantar pressure distribution during barefoot walking. The participants walked three times barefoot over the platform at a self-selected gait speed. A foot mask with five regions of interest (ROI) (rearfoot, midfoot, forefoot, hallux and toes) was applied to assess the maximum force (% Body Weight - BW), peak pressure (kPa), contact area (cm^2^), contact time (ms), pressure-time integral ((kPa)·s), and force-time integral (% BW·s) per ROI. The average of the three trials was used for analysis.

*Tactile Sensitivity*

Tactile sensory deficits were assessed using a 10 g monofilament in four plantar areas (plantar surface of the hallux and heads of the 1st, 3rd, and 5th metatarsals) in both feet, which were tested in randomized order [36,37]. The number of areas where the participant did not feel the pressure applied by the monofilament was recorded [38]. This method demonstrated a moderate reliability by the intraclass correlations between assessors (ICC_(2,3)_ > 0.73) [39].

The tactile sensory threshold was assessed on the dorsal surface of the hallux by using six monofilaments with different degrees of stiffness. Each sensitivity threshold value was transformed into a specific numerical value: 0.05 g, 0.2 g, 2 g, 4 g, 10 g, 300 g, and no sensitivity were represented by 1, 2, 3, 4, 5, 6, and 7, respectively. Both feet were evaluated. Monofilaments were applied in order of increasing stiffness. A positive threshold was recorded when the subject could feel the pressure applied by the filament [40]. This method demonstrated a moderate reliability by the intraclass correlations between assessors (ICC_(2,3)_ > 0.55) [39].

*Passive Ankle Range of Motion*

The passive ankle joint range of motion was evaluated bilaterally by using an ankle electrogoniometer (model SG110/A, Biometrics, Gwent, UK). A forward motion of the lower segment was regarded as flexion (negative values) and a backward motion was regarded as extension (positive values) [41]. After setting the zero angle (90 degrees of the ankle joint flexion angle while lying)**,** the assessor measured the passive range of motion of the participant in supine position. This method demonstrated a moderate, good and poor reliability by the intraclass correlations between assessors (ICC_(2,3)_ > 0.60 (*flexion* right foot); 0.84 (*flexion* left foot); 0.00 (*extension* right foot); 0.41 (*extension* left foot)), respectively [39].

*DPN Symptoms*

The participants answered the Brazilian version of the Michigan Neuropathy Screening Instrument (MNSI) [42]. This questionnaire includes 15 items on the sensitivity of the legs and feet. The confirmatory answers for questions 1, 2, 3, 5, 6, 8, 9, 11, 12, 14 and 15 received a score of 1. A negative answer for questions 7 and 13 also scored 1. Question 4 measures circulatory deficit and question 10 measures general asthenia, and both were not included in the scoring. The total scores therefore ranged from 0 to 13 (13 representing the worst DPN condition).

*Quality of Life*

The participants answered the EuroQoL 5-dimensions (EQ-5D) questionnaire [43], which is a generic instrument used to measure the health-related quality of life and allows an assessor to generate an index representing an individual’s health status. It is based on a classification system that describes health in five dimensions: mobility, personal care, usual activities, pain/discomfort and anxiety/depression. The EQ-5D associates a value between −0.59 and 1.00, which represents the health status of an individual (1.00 being the best possible health condition).

*Foot Health and Functionality*

This study used the Brazilian-Portuguese version of a foot health status questionnaire (FHSQ-BR) translated and validated by Ferreira et al. (2008) [44]. Section I evaluates foot health in four domains: foot pain, foot function, footwear, and general foot health. Section II consists of questions with answer options written in affirmative sentences, along with their corresponding numerical value. Section III collects general demographic data. This study used the scores from Section I only because Section II refers to general health. Each domain was scored from 0 to 100 points, wherein 100 represents the best possible condition and 0 represents the worst condition. The scores were calculated using FHSQ software, version 1.03 (Care Quest, Australia).

### 2.6. Statistical Analysis 

According to some authors, the analysis in any type of a pilot or feasibility study should be primarily descriptive [45] and may focus on estimating the confidence interval [46]. Pilot and feasibility studies are treated as independent studies, and whether they should be analyzed using hypothesis testing is controversial [47,48]. Given that it is inappropriate to assign undue significance to the results of hypothesis testing as no formal calculation of power was performed in these studies, such studies should not be analyzed using hypothesis testing. When a sample size is small, it is likely that an imbalance exists in the pre-randomization covariates, which would require adjustments to the analysis. In addition, the confidence interval is likely inaccurate, even when significant differences exist. The results of any hypothesis testing should therefore be treated as preliminary and must be interpreted with caution, and within-group analyses should therefore be favored. We therefore focused on intra-group comparisons (paired t tests) and reported the mean or median differences and 95% confidence interval.

Baseline assessment outcomes between study groups were compared by Mann–Whitney tests when data were non-normally distributed (Shapiro–Wilk test *p* > 0.05) and by independent t-tests when data were normally distributed. Comparisons between assessments (baseline and T12) within each group were done using paired t-tests for toe and hallux strength and for all plantar pressure variables. In dealing with bilateral data from two legs, conceptual problems led to the recommendation against pooling of data in most situations in foot and ankle research [49]. According to Menz (2004) [49], and given that DPN is a symmetrical disease [1], we chose one side for analysis by randomly selecting a single foot (i.e., the right foot) for biomechanical analysis 

For the clinical data, given their non-normal distribution, the following variables were compared between assessments and within groups using Wilcoxon tests: tactile sensitivity and threshold, FHSQ function and shoes and health domains. The remaining variables (age, body mass, height, body mass index, DPN severity fuzzy score, ankle range of motion (ROM), MNSI, FHSQ pain, EQ-5D, gait speeds and number of steps) were compared between assessments within groups using paired t-tests. The adopted alpha was 0.05.

## 3. Results 

The groups did not significantly differ in any of the outcomes at the baseline assessment (Table 1).

### 3.1. Feasibility Outcomes

#### 3.1.1. Recruitment 

In the first year of recruitment (52 weeks) by using digital advertisements and outpatient clinic databases and via direct contact with people with diabetes through the health campaigns at the university campus, we identified 1549 people with diabetes whose ages fell within the age range set for this study. These individuals were further screened for eligibility by telephone interview (Figure 1, part 1). A total of 144 (9.3%) people were initially found to be eligible for the subsequent laboratory screening based on the inclusion and exclusion criteria, and were willing to participate (Figure 1, part 2). Based on the laboratory screening results, 99 of these 144 potential participants failed to satisfy the eligibility criteria, mainly the requirement for having a moderate or severe DPN. The 45 other participants (31%) were confirmed eligible; thus, the recruitment rate was 0.9 participants/week.

Of the 45 eligible individuals, 30 were included in the baseline assessment (16 males and 14 females), resulting in a recruitment success rate of 66%; for the 15 other individuals, some could not attend the baseline assessment within the period of this feasibility study due to their unavailability, whereas the others provided no reason. The number of participants who were scheduled for the baseline assessment out of the total number of individuals screened within a fixed period (52 weeks) indicated a “successful recruitment,” and the figure is a better predictor of the number of individuals that must be recruited to reach the desired number of included subjects. Based on the 66% recruitment success rate, for a full-blown RCT, 119 individuals must be screened in order to reach the desired number of included participants, which is 77.

#### 3.1.2. Adherence to the Exercise Program and to the Assessments and Dropout Rate

Out of the 15 intervention group participants, 3 (20%) failed to complete at least 80% of the 24 supervised training sessions, that is, the mean adherence was 80%. None of the 30 participants withdrew from this study (0% dropout), and the adherence to the final assessments at T12 was 100%. 

The reported reasons for not joining a supervised training session included a conflicting schedule for hospital-related appointments and the unavailability of a family member who will take the participant to the supervised session. Whenever a participant missed a scheduled session, we rescheduled the session within the same week.

#### 3.1.3. Participant Satisfaction

**Figure 2:**
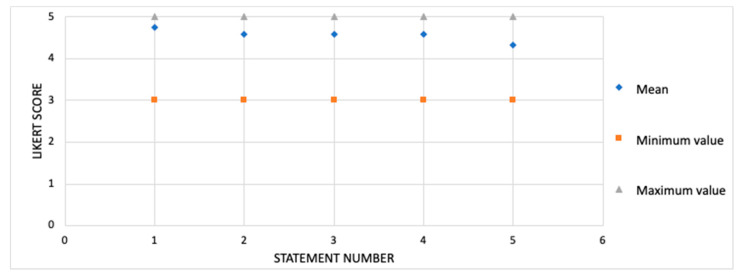
Participant satisfaction with the exercise protocol (n = 15). Scores are shown on a 5-point Likert scale. Data are shown as mean, maximum and minimum. Statement number: (1) “I am satisfied with the exercise protocol”; (2) “The exercise protocol is easy to perform ”; (3) “The exercise protocol is fun to perform”; (4) “The exercise protocol met my expectations”; (5) “The exercise protocol somehow improved my walking practice”.

Overall, the average score for the participants’ satisfaction with the therapeutic foot and ankle exercise program was 4.6 (SD 0.70) on a 5-point Likert scale (Figure 2).

### 3.2. Program Efficacy Outcomes

The MNSI score significantly decreased from the baseline to T12 in both the intervention (*p* = 0.049) and control (*p* = 0.023) groups. Moreover, the FHSQ foot pain score improved in both the intervention (*p* = 0.046) and control (*p* = 0.033) groups (Table 2 and Table 3). In the intervention group, the maximum toe strength significantly increased from the baseline to T12 (*p* = 0.001), a pattern not observed in the control group (*p* = 0.668) (Table 3 and Table 4). 

In the intervention group, the contact time for the toes after 12 weeks of exercise training increased significantly compared with the baseline (*p* = 0.025, Table 4). Additionally, the forefoot peak pressure (*p* = 0.001) and the pressure-time integral (*p* = 0.006) significantly increased in the intervention group. In the control group, the midfoot pressure-time integral significantly decreased (*p* = 0.047), the maximum normalized heel force significantly increased (*p* = 0.049), and the heel peak pressure significantly increased (*p* = 0.049) at T12 compared with the baseline. No other significant time effects were observed in the study groups.

## 4. Discussion

Researchers have recognized that research on the efficacy of interventions can be accelerated if careful feasibility and pilot studies assessing the preliminary efficacy of certain interventions are conducted prior to conducting large RCTs [50]. We therefore report on the feasibility and preliminary efficacy of an ongoing RCT on the effect of a therapeutic foot-ankle exercise program for the biomechanical and clinical outcomes in people with DPN. The results confirmed that this study is feasible based on the 12-week adherence to the assessments and to the intervention protocol at 100% and 80%, respectively; in addition, the participants in the intervention group reported a high satisfaction rate toward the intervention (mean score of 4.57 out of 5). However, the recruitment rate was low at 0.9 patients/week compared with the 10 patients/week rate that was possible considering the availability of a laboratory. This low recruitment was mainly due to the rigorous eligibility criteria, and new strategies for improving recruitment must be employed to reduce the recruitment time for RCT completion. As regards the preliminary efficacy, among all the functional, biomechanical and clinical outcomes, a few were significantly improved in the intervention group when the baseline and T12 were compared; these outcomes were toe strength, DPN symptoms and specific plantar pressure parameters. This finding may help in the study design or in the analysis of the efficacy of foot-ankle exercise programs in large RCTs. 

### 4.1. Feasibility

The recruitment rate was low (0.9 patients/week) mainly because many patients did not meet the eligibility criterion “severe and moderate DPN” according to their fuzzy scores. Thus, new recruitment strategies were developed to improve the recruitment rate and to guarantee the success of the RCT recruitment. A partnership with the largest hospital in Latin America (Hospital das Clínicas da Faculdade de Medicina da USP) was entered into, providing us access to a database of approximately 4000 people with diabetes whom we could recruit for our RCT. Based on the recruitment success rate of 66%, 119 initially eligible subjects are needed to include 78 participants for the RCT, which is achievable using the new database. Although 66% is a moderate rate, the achieved recruitment success rate is compatible with several other operational factors that influence the flow of assessments and intervention; such factors include the period the biomechanics laboratory facilities were operational, the time spent on each biomechanical assessment, and the strategy employed to avoid scheduling follow-up assessments and baseline assessments within the same week.

The participants were satisfied with the exercise therapeutic program as shown by the mean score of 4.57 based on a 5-point scale. In general, foot-ankle exercise programs are well-accepted [51]. This outcome seems directly related to adherence; an 80% adherence can be considered high and it falls within the range that indicates feasibility [52]. Moreover, the high satisfaction with the training program was apparently reflected in the 0% dropout from the 12-week study, suggesting that the chosen exercise protocol is appropriate for further investigation in the RCT. A previous trial [53] did not observe any effect of a 24-week intervention that combined lower limb strengthening and gait and balance training, probably due to lack of motivation and a high dropout rate (42%), which may have been influenced by the lack of an exercise progression that was not tailored according to the progression made by the participant, different to what we implemented in the present RCT.

### 4.2. Program Efficacy

The signs and symptoms of DPN had changed after the intervention (MNSI score). The intervention group reported fewer symptoms after the intervention compared with their baseline condition. In the control group, the MNSI score slightly improved after 12 weeks. There are some possible explanations for the improvement of symptoms in both groups. First, there could be a placebo effect resulting from the interaction of the physiotherapist with the participants, which may bring about a positive response independent of any specific treatment [54]. Second, the usual care orientations given by the physiotherapist to both groups would lead to changes in the participant´s health care habits that could result in a better control of diabetes and thus of the DPN symptoms. Lastly, within 12 weeks, there could be a natural variation in the DPN symptoms not related to any intervention effect [10].

After 12 weeks of the foot-ankle exercises, only the toe strength of all the functional outcomes significantly increased with a mean of 1.6% BW, representing a 27% increase in isometric strength. The exercise protocol focused on strengthening several flexor muscles of the interphalangeal and metatarsalphalangeal joints, including the flexor digitorum brevis and longus, flexor digiti minimi brevis, quadratus plantae, lumbricals, plantar and dorsal interossei muscles of the toes. We presume that this result is directly related to the foot-ankle exercise program. Our result was slightly lower than that reported by Mickle et al. (2016) wherein the increase in toe flexor strength in older adults after 12 weeks of exercise was 36% [55]. Another study showed that exercises promoting the foot muscle strength in young runners significantly increased the toe-flexor muscle strength by 16% after 5 weeks of exercise and by 27% after 10 weeks [56]. Unfortunately, in clinical contexts, a certain degree of unwillingness to accept the prescription of foot-ankle exercises in DPN patients has been observed because it is widely believed that muscle weakness and joint limitations are irreversible in DPN. Our results suggest that this is not the case, consistent with other findings showing that, despite the low level of evidence [23], foot-ankle exercises improve the muscle function and joint mobility in people with DPN [10,12,13,14,15,21,22]. A larger RCT should confirm whether this finding establishes a foundation for the implementation of physical therapy intervention for neuromuscular diseases, including DPN.

The intervention did not yield many changes in plantar pressure distribution after 12 weeks: the toe contact time was prolonged and the forefoot peak pressure and pressure-time integral were increased. The former suggests that the intervention might have increased the contribution of the toes during a foot rollover, mainly during body propulsion, leading to a prolonged contact and higher/prolonged pressures applied to the ground. This finding is important as the contribution of the toes is reduced during locomotor tasks in patients with DPN [6], and this phenomenon is usually attributed to restrictions in foot-ankle joint mobility [57,58] and to intrinsic muscle weakness [59].

The increased forefoot pressures may have been due to the changing role of the forefoot in gait propulsion given that the intervention focused on improving the intrinsic foot muscle strength and function. These findings suggest that while attention must be devoted in keeping plantar pressures below the risk threshold for ulceration in people with DPN [60], this should not be the sole aim of physiotherapeutic interventions, as the results showed that the foot-ankle exercise programs demonstrated several beneficial effects in the investigated population. Within 12 weeks, the control group showed an increased mean force and peak pressure in the heel, worsening the distribution of pressure over the foot and probably the foot rollover over time in the absence of any specific intervention. The strengthening exercises and walk training demonstrated a preliminary efficacy in improving toe function as reflected in foot rollover changes, which we believe is a promising path toward maintaining the residual biomechanical capability of propelling the body forward during walking; however, a larger RCT should provide more sound evidence to support these preliminary effects.

The biomechanical sensor−pressure plate that was used to measure the effect of the intervention on foot isometric strength and plantar pressure distribution during gait has shown its potential to identify changes over time in the studied population and could be an indicator of great value of the improvement of DPN due to a therapeutic foot-ankle exercise program. Data from pressure sensors were the primary outcome in another trial focusing on the effectiveness of foot-ankle exercises in individuals with DPN, and the authors observed a change in the foot rollover towards a more physiological process, supported by the improved plantar pressure distribution measured [10]. Although data derived from pressure plates have been recognized as an important parameter to determine the onset of diabetic foot ulcers, the information from plantar pressure sensors is not used on a regular basis in clinical settings to diagnose and manage impairments associated with various musculoskeletal, integumentary, and neurological disorders [61], such as DPN. Our results showed that pressure data could potentially contribute to tailoring therapeutic strategies for people with diabetes and DPN and monitoring the short- and long-term effects of exercises intervention on gait biomechanics.

Although wearable sensors (accelerometers) similar to those selected to assess the level of physical activity in our study have long been recognized to be one of the most effective ways to objectively measure physical activity, they have been widely used in controlled conditions [62]. In our feasibility study, we used this sensor in real life, aiming to quantify the physical activity by counting the number of steps during a regular week of the participant. We believe that this measure is a strong indicator of physical heath, especially in DPN individuals. For instance, average daily steps count in people with diabetes and DPN was found to be inversely related to intermuscular adipose tissue volume [63]. 

The strengths of this study include the rigorous RCT methodology, that is, a supervised physiotherapeutic approach combined with remote intervention, which we think has increased the participant satisfaction and adherence and contributed to the low dropout rate. For these reasons, the intervention was considered feasible and no further amendments will be made in the trial registry and protocol. We are the first to evaluate the musculoskeletal outcomes in people with DPN through a specialized foot training protocol that focuses on improving the strength and functionality of the foot-ankle complex. A limitation of this study has to do with recruitment. Despite the high prevalence of diabetes in the Brazilian population [64], the recruitment rate for this RCT was low due to the rather strict eligibility criteria. In addition, the two study groups did not experience the same degree of on-site interaction; the control participants had an interaction with the physiotherapist and received feedback only during the baseline and 12-week assessments, whereas the intervention group participants had weekly interactions with the physiotherapist. This difference might have led to a greater degree of dissatisfaction toward the study, and it may result in a greater dropout rate among control subjects in a larger trial. Furthermore, other usual parameters related to the clinical control of diabetes, such as hemoglobin glycade and glycaemia, were not assessed and might have influenced the investigated functional and clinical outcomes. Other aspects of the training, such as nocebo or placebo effects, might also be relevant factors that may have obscured the genuine effects of the training program.

## 5. Conclusions

We conclude that this study is feasible based on moderate recruitment and on the adherent and satisfied study population; thus, no further amendments in the protocol and trial are needed. The foot-ankle exercise program showed some positive preliminary clinical, functional and biomechanical effects over time, such as an improvement in strength and mobility of people with DPN, which justifies the further assessment of outcomes in a larger RCT.

## Figures and Tables

**Table 1:** Baseline participants’ characteristics from the control and intervention groups.

Outcomes	Control Group (n = 15)	Intervention Group (n = 15)	*p*-Value
Age (years)	62.5 (6.8)	64.6 (6.9)	0.220 ^1^
Body mass (kg)	78.4 (17.5)	78.6 (20.0)	0.485 ^1^
Height (m)	1.6 (0.1)	1.7 (0.1)	0.178 ^1^
Body Mass Index (kg/m^2^)	28.9 (5.3)	28.1 (7.0)	0.364 ^1^
Type of diabetes	DM1 = 0% DM2 = 100%	DM1 = 33.3% DM2 = 66.7%	0.063 ^3^
Sex	M-7 F-9	M-9 F-5	1.000 ^3^
DPN severity (Fuzzy Score)	4.4 (2.2)	5.6 (3.0)	0.105 ^1^
MNSI (score)	6.1 (2.2)	6.3 (3.8)	0.816 ^1^
Tactile sensitivity (number of areas)	2.4 (2.4)	3.3 (2.9)	0.445 ^2^
Tactile sensitivity threshold right	3.0 (2–7) ^¶^	3.0 (2–7) ^¶^	1.000 ^2^
Tactile sensitivity threshold left	3.0 (2–7) ^¶^	3.0 (2–7) ^¶^	1.000 ^2^
Self-selected gait speed (m/s)	1.0 (0.2)	1.1 (0.4)	0.478 ^1^
Fast gait speed (m/s)	1.5 (0.4)	1.6 (0.3)	0.694 ^1^
Daily activity level (number of steps)	8134.6 (5055.2)	7810.8 (4268.3)	0.844 ^1^
FHSQ pain (score)	58.7 (24.6)	54.2 (35.8)	0.651 ^1^
FHSQ function (score)	70.4 (25.8)	72.9 (30.6)	0.600 ^2^
FHSQ shoes (score)	39.4 (33.7)	48.9 (41.8)	0.501 ^2^
FHSQ health (score)	37.5 (31.2)	32.5 (23.0)	1.000 ^2^
EQ-5D (score)	0.4 (0.2)	0.4 (0.2)	0.581 ^1^
Ankle dorsiflexion ROM right (°)	19.0 (5.8)	16.6 (7.7)	0.555 ^1^
Ankle dorsiflexion ROM left (°)	21.7 (7.5)	17.3 (6.2)	0.215 ^1^
Ankle plantarflexion ROM right (°)	25.7 (8.4)	28.67 (10.0)	0.331 ^1^
Ankle plantarflexion ROM left (°)	30.5 (8.7)	31.9 (9.8)	0.883 ^1^

Data are presented as mean (SD) or as n or %. ^¶^ Mode (minimum–maximum range). ^1^ t-test; ^2^ Mann–Whitney test; ^3^ Chi-square test. MNSI- Michigan neuropathy Screening Instrument questionnaire.

**Table 2:** Clinical outcomes and foot health and functionality of each group (control and intervention).

Outcomes	Control Group		Control Effect		Intervention Group		Intervention Effect	
	Baseline (n = 15)	T12 (n = 15)	*p* Value	Difference (CI 95%)	Baseline (n = 15)	T12 (n = 15)	*p* Value	Difference (CI 95%)
MNSI questionnaire (mean Score) ^1^	6.1 (2.0)	4.9 (3.1)	0.023 *	1.2 (0.1 to 2.1)	6.3 (3.8)	5.2 (3.1)	0.049 *	1.1 (−0.0 to 2.3)
Tactile sensitivity (number of areas) ^2^	2.4 (2.4)	2.7 (2.7)	0.559	−0.3 (−1.5 to 0.9)	3.2 (2.9)	3.0 (2.6)	0.739	0.2 (−1.0 to 1.4)
Tactile sensitivity threshold Right ^2^	3.0 (2.0–7.0)^τ^	3.0 (1.0–7.0)^τ^	0.957	-	3.0 (2.0–7.0)^τ^	3.0 (2.0–7.0)^τ^	1.000	-
Tactile sensitivity threshold Left ^2^	3.0 (2.0–7.0)^τ^	3.0 (1.0–7.0)^τ^	1.000	-	3.0 (2.0–7.0)^τ^	3.0 (2.0–7.0)^τ^	1.000	-
EQ-5D questionnaire (Score) ^1^	0.36 (0.1)	0.40 (0.1)	0.352	-0.04 (-0.14 to 0.06)	0.36 (0.1)	0.41 (0.2)	0.161	−0.05 (−0.10 to 0.02)
FHSQ—foot pain (Score) ^1^	58.7 (24.6)	66.3 (23.0)	0.033 *	−7.6 (−14.5 to −0.7)	54.2 (35.7)	68.9 (23.6)	0.046 *	−14.7 (−29.9 to 0.5)
FHSQ—foot function (Score) ^2^	70.4 (25.8)	69.7 (23.2)	0.888	0.7 (−9.7 to 11.1)	72.9 (30.5)	79.2 (26.1)	0.181	−6.3 (−15.8 to 3.3)
FHSQ—shoes (Score) ^2^	39.4 (33.6)	40.1 (34.8)	0.902	−0.7 (−12.2 to 10.8)	48.9 (41.1)	42.2 (39.2)	0.417	6.7 (−10.4 to 23.8)
FHSQ—foot health (Score) ^2^	37.5 (31.2)	46.0 (26.0)	0.089	−8.5 (−18.5 to 1.5)	32.5 (23.0)	44.2 (21.4)	0.097	−11.7 (−25.8 to 2.4)

Data are presented as mean (SD) and mean differences with 95% confidence intervals (CI). ^1^ *p* values related to paired t-tests, ^2^ *p* values related to Wilxocon matched pairs. * and bold *p* values represents difference between baseline and T12 within the group. ^τ^ represents mode (minimum–maximum). MNSI—Michigan Neuropathy Screening Instrument, FHSQ—foot health status questionnaire.

**Table 3:** Functional outcomes of each group (control and intervention).

Outcomes	Control Group		Control Effect		Intervention Group		Intervention Effect	
	Baseline (n = 15)	T12 (n = 15)	*p* Value	Mean Difference (CI 95%)	Baseline (n = 15)	T12 (n = 15)	*p* Value	Mean Difference (CI 95%)
Ankle ROM dorsiflexion right (°)	19.0 (5.7)	16.7 (6.1)	0.214	2.3 (−1.5 to 6.0)	16.6 (7.1)	19.3 (6.1)	0.137	−2.7 (−6.4 to 0.98)
Ankle ROM dorsiflexion left (°)	21.7 (7.5)	18.5 (5.0)	0.113	3.2 (−0.8 to 7.2)	17.3 (6.2)	18.0 (4.8)	0.637	−0.7 (−3.6 to 2.3)
Ankle ROM plantarflexion right (°)	25.7 (8.4)	29.9 (7.4)	0.019	−4.2 (−7.7 to −0.8)	28.7 (9.9)	28.8 (7.3)	0.947	−0.1 (−4.4 to 4.1)
Ankle ROM plantarflexion left (°)	30.5 (8.7)	30.9 (13.4)	0.701	−0.4 (−3.03 to 2.10)	31.9 (9.7)	32.0 (7.5)	0.951	−0.1 (−4.7 to 4.4)
Self-selected gait speed (m/s)	1.03 (0.23)	1.02 (0.31)	0.986	0.01 (−0.16 to 0.16)	1.14 (0.36)	1.06 (0.16)	0.342	0.08 (-0.10 to 0.25)
Fast gait speed (m/s)	1.50 (0.38)	1.44 (0.35)	0.444	0.06 (−0.40 to 0.23)	1.56 (0.33)	1.70 (0.44)	0.142	−0.14 (−0.30 to 0.05)
Number of steps for 6 days	8135 (5055)	7280 (3393)	0.367	854 (−1110 to 2819)	7811 (4268)	9137 (4964)	0.337	−1326 (−4189 to 1536)
Maximum force—hallux (%BW)	10.8 (3.8)	9.6 (4.3)	0.368	1.2 (−1.5 to 3.9)	11.8 (5.9)	12.1 (6.0)	0.727	−0.3 (−2.0 to 1.4)
Maximum force—toes (%BW)	7.5 (4.3)	7.2 (4.0)	0.668	0.3 (−1.2 to 1.8)	6.4 (2.8)	8.9 (4.0)	0.001 *	−2.5 (−3.8 to 1.2)
Maximum force—all toes (%BW)	11.3 (3.4)	10.8 (4.1)	0.676	0.5 (−2.1 to 3.1)	12.0 (5.9)	13.2 (4.8)	0.161	−1.2 (−3.1 to 0.6)

Data are presented as mean (SD) and mean differences with 95% confidence intervals (CI). *p* values related to paired t-tests. * and bold *p* values represents difference between baseline and T12 within the group. BW—body weight; ROM—range of motion.

**Table 4:** Plantar pressure distribution variables during gait of each group (control and intervention).

Plantar Pressure During Gait									
Region of Interest	Parameters	Control Group		Control Effect		Intervention Group		Intervention Effect	
		Baseline (n = 15)	T12 (n = 15)	*p* Value	Difference (CI 95%)	Baseline (n = 15)	T12 (n = 15)	*p* Value	Difference (CI 95%)
Toes	CA [cm^2^]	9.3 (4.0)	9.1 (3.8)	0.675	0.2 (−0.9 to 1.3)	7.4 (3.2)	8.1 (3.0)	0.291	−0.7 (−2.2 to 0.7)
	MF [%BW]	6.3 (3.7)	6.7 (4.1)	0.523	−0.4 (−1.9 to 0.9)	6.3 (5.7)	6.0 (3.8)	0.751	0.3 (−1.6 to 2.1)
	PP [kPa]	174 (111)	174 (103)	0.994	0.1 (−37.1 to 37.4)	268 (172)	244 (136)	0.494	24.0 (−50.1 to 98.2)
	CT [ms]	562 (162)	501 (124)	0.060	61.2 (8.9 to 113.6)	519 (119)	578 (58)	0.025 *	−59.2 (−122.4 to 3.9)
	PTI [(kPa)·s]	58.0 (44.4)	50.8 (32.8)	0.279	7.2 (−6.7 to 21.0)	64.3 (47.0)	73.7 (35.2)	0.233	−9.4 (−25.8 to 6.9)
	FTI [%BW·s]	1.9 (1.4)	1.8 (1.2)	0.541	0.1 (−0.4 to 0.7)	1.5 (1.1)	1.7 (0.9)	0.346	−0.2 (−0.6 to 0.2)
Hallux	CA [cm^2^]	9.5 (2.3)	9.7 (2.2)	0.655	−0.2 (−1.2 to 0.8)	10.0 (2.2)	10.9 (2.2)	0.073	−0.9 (−1.6 to 0.08)
	MF [%BW]	11.9 (7.4)	12.2 (5.8)	0.789	−0.3 (−2.6 to 1.9)	16.9 (9.4)	15.1 (8.7)	0.511	1.8 (−2.3 to 4.3)
	PP [kPa]	297 (246)	291 (232)	0.859	6.1 (−66.8 to 7.9)	415 (274)	424 (273)	0.766	−9.0 (−118.7 to 89.8)
	CT [ms]	512 (201)	493 (182)	0.587	19.1 (−55.5 to 93.7)	526 (141)	571 (151)	0.371	−45.2 (−159.6 to 64.6)
	PTI [(kPa)·s]	90.8 (92.1)	86.2 (91.2)	0.624	4.6 (−15.3 to 24.6)	112.5 (96.3)	122.5 (83.0)	0.456	−10.0 (−60.6 to 29.1)
	FTI [%BW·s]	3.5 (3.1)	3.4 (2.8)	0.786	0.1 (−0.7 to 0.9)	3.8 (2.5)	4.1 (2.3)	0.331	−0.3 (−1.5 to 0.5)
Forefoot	CA [cm^2^]	52.9 (9.9)	53.5 (10.2)	0.302	−0.6 (−1.7 to 0.6)	48.6 (7.3)	49.0 (7.0)	0.485	−0.3 (−1.4 to 0.7)
	MF [%BW]	103.3 (7.3)	106.7 (9.5)	0.070	−3.4 (−7.2 to 0.4)	98.2 (11.6)	102.2 (6.3)	0.194	−4.0 (−10.2 to 2.3)
	PP [kPa]	709 (202)	771 (254)	0.090	−62.5 (−136.6 to 11.6)	790 (273)	959 (244)	0.001 *	−169.1 (−225.2 to −82.7)
	CT [ms]	736 (92)	704 (110)	0.134	31.3 (−11.2 to 73.7)	698 (131)	711 (94)	0.589	−12.8 (−63.1 to 37.5)
	PTI [(kPa)·s]	255.2 (74.2)	262.8 (104.8)	0.652	−7.6 (−43.6 to 28.3)	302.1 (146.3)	365.4 (160.0)	0.006 *	−63.3 (−105.5 to −21.2)
	FTI [%BW·s]	41.6 (6.2)	40.7 (6.4)	0.482	0.9 (−1.8 to 3.7)	37.0 (7.9)	40.2 (6.9)	0.056	−3.2 (−6.5 to 1.1)
Midfoot	CA [cm^2^]	30.3 (8.2)	30.2 (8.1)	0.987	−0.0 (−0.9 to 0.8)	26.7 (9.3)	27.4 (9.9)	0.404	−0.7 (−2.5 to 1.1)
	MF [%BW]	27.0 (8.2)	26.1 (7.5)	0.393	0.9 (−1.4 to 3.3)	25.1 (15.2)	26.8 (17.0)	0.269	−1.7 (−4.9 to 1.5)
	PP [kPa]	167 (63)	166 (54)	0.863	0.9 (−10.9 to 12.9)	233 (155)	291 (229)	0.231	−57.6 (−156.8 to 41.7)
	CT [ms]	577 (105)	561 (122)	0.448	16.1 (−28.5 to 60.5)	594 (100)	569 (111)	0.464	25.2 (−47.3 to 97.5)
	PTI [(kPa)·s]	68.6 (28.5)	62.2 (24.8)	0.047 *	6.4 (0.1 to 12.7)	80.4 (56.8)	90.7 (53.4)	0.430	−10.3 (−37.8 to 17.2)
	FTI [%BW·s]	9.3 (3.2)	8.2 (2.7)	0.052	1.1 (−0.0 to 2.2)	8.9 (6.9)	9.0 (6.0)	0.773	−0.1 (−1.6 to 1.2)
Heel	CA [cm^2^]	34.6 (5.1)	35.0 (4.5)	0.248	−0.4 (−1.1 to 0.3)	34.0 (5.1)	33.8 (5.4)	0.684	0.2 (−0.7 to 1.0)
	MF [%BW]	63.1 (10.7)	68.5 (8.7)	0.049*	−5.4 (−11.2 to 0.3)	71.0 (19.3)	66.7 (17.2)	0.271	4.3 (−3.8 to 12.4)
	PP [kPa]	392 (193)	459 (261)	0.049 *	−66.7 (−133.2 to −0.3)	441 (165)	455 (146)	0.551	−14.2 (−64.7 to 36.2)
	CT [ms]	513 (83)	500 (137)	0.640	12.6 (−44.5 to 69.7)	467 (194)	482 (105)	0.704	−15.4 (−101.7 to 70.9)
	PTI [(kPa)·s]	103.7 (40.9)	118.5 (78.6)	0.294	−14.8 (−44.2 to 14.6)	110.3 (39.0)	111.7 (41.4)	0.828	−1.4(−14.8 to 12.1)
	FTI [%BW·s]	17.5 (3.9)	18.6 (4.3)	0.362	−1.1 (−3.4 to 1.3)	19.1 (5.6)	17.6 (5.9)	0.288	1.5 (−1.4 to 4.4)

Data are presented as mean (SD) and mean differences with 95% confidence intervals (CI). * and bold *p* values represents difference between baseline and T12 within the group. *p* values related to paired t-test. CA—contact area; MF—maximum normalized force; PP—peak pressure; CT—contact time; PTI—peak-time integral; FTI—force-time integral.
